# Rapid Cycle Deliberate Practice Compared With Traditional Simulation Debriefing for Resuscitation Skills Training in Pediatric Residents

**DOI:** 10.7759/cureus.97096

**Published:** 2025-11-17

**Authors:** Shannon M Flood, Matthew Mittga, Jonathan Higgins, Laura Rochford, Brad Sobolewski, Aya Angstadt, Erin McGonagle, Mairead Dillon, Lilliam Ambroggio, Kathryn Walsh

**Affiliations:** 1 Pediatric Emergency Medicine, University of Colorado, Aurora, USA; 2 Pediatric Emergency Medicine, Children's Hospital Colorado, University of Colorado School of Medicine, Aurora, USA; 3 Pediatric Emergency Medicine, Cincinnati Children's Hospital, Cincinnati, USA; 4 Pediatric Emergency Medicine, University of Colorado School of Medicine, Aurora, USA; 5 Pediatric Critical Care Medicine, University of Colorado, Aurora, USA

**Keywords:** paediatric resuscitation, pediatric resident training, randomized trial, rapid cycle deliberate practice (rcdp), simulation in medical education

## Abstract

Background: Rapid cycle deliberate practice (RCDP) is a form of simulation debriefing that incorporates repeated cycles of hands-on practice, characterized by within-simulation directed feedback and repeated practice with the goal of mastering a skill. RCDP debriefing has been shown to improve immediate performance; however, evidence of improved retention and superiority to traditional styles of debriefing is lacking.

Objectives: To compare RCDP and traditional debriefing for knowledge and skills acquisition and retention as part of a longitudinal residency simulation curriculum.

Design: Participants were first-year pediatrics residents who underwent two simulation scenarios, focusing on basic airway and cardiac arrest management, at the beginning of their intern year. Participants were block randomized to either the RCDP or the traditional debriefing arm. Knowledge was assessed in a pre-post format at time 0 and at 12 months using a multiple-choice quiz. All interns received a repeat simulation teaching session at three, six, nine, or 12 months, remaining in their RCDP or traditional debriefing arm. Skills were assessed via a video-recorded simulated scenario prior to an initial simulation session (RCDP vs. traditional debriefing) at time 0 and three, six, nine, and 12 months, and before and after their repeat simulation teaching session. Videos were scored by two pediatric emergency medicine physicians using a resuscitation skills assessment tool.

Results: There was no statistical difference in overall knowledge (via paired t-test) or skills acquisition (via linear mixed effects model) between residents who received traditional debriefing and those who received RCDP debriefing. The RCDP group showed significant improvement in skills when compared with the traditional group for those who received repeat education at three months; however, there was no difference in the other time groups. There was no difference between groups in skills retention at three months after the repeat teaching session, as assessed via the ANOVA analysis.

Conclusion: Overall, we demonstrated similar knowledge and skills gain and retention in traditional debriefing and RCDP groups, with the exception of those residents who received repeat teaching at the three-month time point. This may indicate that a three-month time interval could be the most appropriate timing for repeated RCDP resuscitation teaching. Higher power randomized controlled trials comparing RCDP to traditional simulation and/or qualitative studies assessing the efficacy of RCDP would add to current evidence.

## Introduction

Cardiopulmonary arrest (CPA) in children is a rare but life-threatening condition, occurring 20,000 times annually, and in which mortality rates range from 11.4% for out-of-hospital CPA up to over 40% for in-hospital CPA. Despite its high morbidity and mortality rates, these events are relatively rare at 2.8 per 100,000 person-years [1]. High-risk yet low-frequency events are well-suited for simulation-based medical education to provide education and skills practice outside of live clinical events, which occur infrequently clinically.

Pediatric residency training provides the initial introduction to pediatric-specific cardiopulmonary resuscitation (CPR) education for most medical school graduates. The American Heart Association has established best-practice guidelines and a curriculum for the management of pediatric CPA aimed at improving outcomes and standardizing resuscitation education [1]. Pediatric advanced life support (PALS) training programs represent the primary method for pediatric residency programs to train residents in CPR, and the Accreditation Council for Graduate Medical Education (ACGME) requires maintenance of certification in PALS for all residents in pediatrics programs [2]. Adherence to PALS guidelines during resuscitation has been shown to correlate with improved survival after CPA [1,3-5]. Despite the initial training and every two-year re-certification requirement, adherence to PALS guidelines by pediatric residents is poor [6,7], with most performing poorly on PALS-specific standards for resuscitative care and showing no clear improvement throughout their three years of residency training [6]. Notably, the ACGME recently revised pediatric residency training requirements, removing a number of procedural skills, including some PALS-specific skills. These changes allow programs to reduce the scope of PALS and procedural training [8] and may further reduce residents’ skills upon graduation.

One type of curriculum to address this gap and develop competency in pediatric resuscitation is simulation-based curricula. Simulation-based educational curricula developed to teach resuscitation care have shown promising preliminary results, leading to increased adoption of simulation curricula throughout training programs [9-12]. A simulation teaching technique called rapid cycle deliberate practice (RCDP) is a simulation-based mastery learning program with three core principles: (1) provide learners multiple opportunities to “get it right”; (2) offer in-the-moment, time-sensitive feedback from experts; and (3) maintenance of a psychologically safe environment for learning. RCDP is founded on deliberate practice, developed by K. Anders Ericsson, a theoretical framework that focuses on repetition and successive refinement to improve performance [13]. This style of simulation differs from traditional debriefing, in which participants engage in a simulation scenario uninterrupted, then discuss the experience, focusing on specific learning objectives. Traditional simulation debriefing tends to be more learner-led, allowing participants to guide the discussion, while RCDP is more objective or facilitator-led, with a facilitator in the simulation space, interrupting the scenario to address a specific objective, allowing for repeated practice, and then continuing the scenario. RCDP differs from individual skills mastery or task training education in that it is team-based and used not only for hands-on skills but also for communication skills and team coordination. RCDP has been studied in varying environments and has been shown to improve confidence, knowledge, and skill in simulated resuscitation clinical care [14-18], including improved PALS performance that was sustained at three, six, and nine months [19,20]. RCDP has demonstrated improved short-term knowledge and specific skill retention in simulation; however, it has not been directly compared with a more traditional style of simulation debriefing for longer-term skills retention [21,22]. Existing evidence remains mixed regarding the best method of simulation to teach resuscitation skills and the frequency with which teaching sessions need to be repeated to achieve retention of skills, knowledge, and confidence. Given that RCDP is a resource-intensive form of debriefing, requiring trained simulation educators, facilitators, and technicians in this specific style of debriefing, more data are required before advocating for programs to transition to this style of debriefing. Our primary objective was to compare RCDP with traditional debriefing for acquisition and retention of resuscitation knowledge and skills over time during the first year of pediatric residency training. Our secondary objective was to assess and compare the effect of repeated education with both RCDP and traditional debriefing techniques.

## Materials and methods

Study design

This was a randomized repeated measures study conducted at a single residency program in Colorado from June 2021 to June 2022. The study was approved by the Colorado Multiple Institutional Review Board (Approval No. 20-0657).

Participants

**Figure 1:**
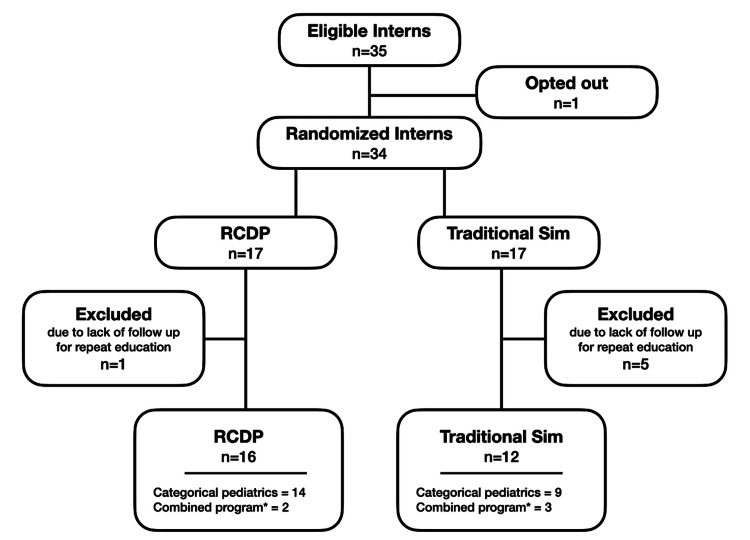
Flow of the study. * Pediatrics/child neurology, pediatrics/genetics, and pediatrics/physical medicine & rehabilitation. RCDP: rapid cycle deliberate practice.

Participants were first-year pediatric residents, also known as interns, including residents in combined training in pediatric neurology and pediatric genetics/metabolic from a tertiary care academic hospital. Internal medicine-pediatrics residents were excluded due to the inability to schedule teaching sessions and concern for additional confounding from adult resuscitation experience. An a priori power calculation was not performed, given that the number of intern participants was fixed; therefore, we used the accessible population. Simulation learning sessions were considered required for the pediatric residency program; however, participation in study procedures, such as filling out surveys before and after the simulation, was voluntary. Simulation sessions with traditional style debriefing were an established portion of the intern curriculum prior to the study. A postcard consent form was used to inform participants of the study via email, and verbal consent was obtained at the initial teaching session. One intern opted out of the study. Interns were excluded from analysis if they did not participate in a post-education assessment. Prior to the onset of residency, interns were provided with a brief simulation experience survey. A total of 28 interns were included in the study at the time of study completion (Figure 1).

Intervention

**Figure 2:**
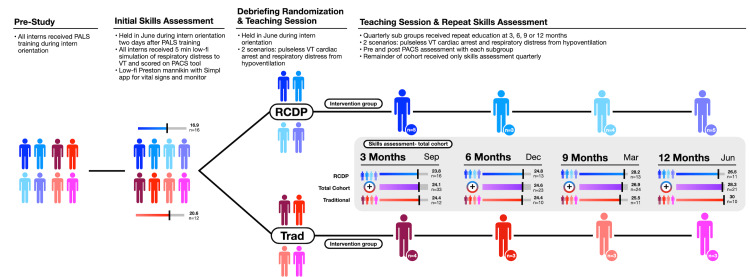
Study design and assessment scores. RCDP: rapid cycle deliberate practice; PALS: pediatric advanced life support; VT: ventricular tachycardia; PACS: pediatric airway and circulation skills.

All simulation learning sessions for both groups in the pediatric simulation center at Children’s Hospital Colorado use Laerdal SimBaby and SimMan manikins (Laerdal Medical, Stavanger, Norway). The initial learning session was conducted during interns’ residency orientation, prior to the commencement of residency rotations, but after a PALS certification course. Prior to the learning sessions, all residents participated in an individual skills assessment simulation scenario with a simulation facilitator managing the scenario. As per recommendations from an RCDP scoping review [23], this represented an uninterrupted simulation session prior to the educational intervention. Assessment scenarios were video-recorded and saved in a password-protected folder throughout the study period (Figure 2).

Equipment utilized during the assessment scenarios included a Zoll defibrillator (ZOLL Medical Corporation, Chelmsford, MA), oxygen tank, training defibrillator pads, and rhythm generator. Basic airway supplies, such as nasal cannulas, oral airways, simple oxygen masks, and bag-valve masks, were provided. After the assessment scenarios, interns participated in the learning session. They were given objectives for the two scenarios, which included (1) basic airway resuscitation techniques, including placement of oxygen via nasal cannula and via simple mask, bag mask ventilation with flow-inflating and self-inflating bags, and placement of a nasopharyngeal airway, and not including placement of an advanced airway, and (2) performing CPR (initiation of chest compressions and coordination with rescue breaths delivered by bag-mask ventilation (BMV)) and use of the Zoll defibrillator (including pad placement and defibrillation). The scenarios that fulfilled these objectives were (1) hypoventilation from tetrahydrocannabinol (THC) ingestion and (2) cardiac arrest secondary to ventricular tachycardia. The same scenarios were used in both the RCDP group and the traditional group.

Interns were block randomized based on their intern orientation schedule to either the RCDP or traditional debriefing arm and received a one-hour-long learning session according to their study arm. All interns returned for recurrent skills assessments at three-month intervals throughout the year (Figure 2). Each intern also had one additional four-hour simulation learning session with the same teaching objectives at either three, six, nine, or 12 months after the initial learning session. The same two scenarios were used in the repeat learning session (hypoventilation and cardiac arrest). When interns returned for their repeat learning session, they participated in a pre and post skills assessment (Figure 2). A repeated measures design was favored over a parallel design to assess differences in acquisition and retention at different time intervals. Timing of repeated education (three, six, nine, or 12 months) was based upon the residents’ clinical schedule; however, they remained in their original RCDP or traditional study arm regardless of the timing of their repeat education session.

The RCDP debriefing method included “hard” and “soft” stops with a trained RCDP facilitator in the room, providing in-the-moment feedback and opportunities for repeated practice. RCDP scenarios were developed and piloted with residents by the two trained RCDP facilitators who facilitated the sessions (SF and KW). RCDP facilitators paused, rewound, and restarted the scenario depending on the team’s performance. RCDP facilitators completed the Johns Hopkins RCDP training online course, and scenarios were created based on a standardized format provided by the Johns Hopkins Simulation Center RCDP training team. The traditional debriefing method allowed the interns to progress through the scenario to completion or a time-defined ending and then verbally debriefed in a separate room. RCDP facilitators remained in the simulation room with the participants throughout the scenario to pause and provide feedback in real time. There were a total of four traditional simulation debrief facilitators, all of whom completed the Center for Medical Simulation debriefing course, who performed the traditional debriefing. A simulation technician controlled the manikin as per the outlined scenario from inside the simulation control room for both the traditional debriefing sessions and the RCDP sessions. Teaching sessions were not video-recorded.

The pediatric airway and circulation skills (PACS) assessment tool was created by the study group for use during the quarterly individual skills assessments. The PACS tool is novel and was created specifically based upon the objectives of the clinical simulation scenarios and learning sessions, which were developed to address specific gaps in our resuscitation curriculum per an internal needs assessment. It has not been formally validated; however, it was based upon the Knowledge and Clinical Skills portion of the Resuscitation Team Leader Evaluation tool by Grant et al. and the PALS assessment tool by Donoghue et al., both of which demonstrated content validity, inter-rater reliability, and generalizability [24,25]. We chose to use a novel tool as opposed to a previously studied tool to be precise in regards to our curricular objectives and to assess individuals as opposed to teams. While there are previously published resuscitation assessment tools with associated validity evidence, these tools did not specifically address gaps highlighted by our needs assessment, and therefore, the team created a tool specific to our curricular objectives. Skills assessments were low fidelity with a Prestan manikin and performed in a treatment room on the hospital ward floor for ease of resident scheduling and attendance. Skill assessments were video-recorded with an iPhone, after which the videos were transferred to password-protected files. Video-recorded individual assessments were viewed and scored by two pediatric emergency medicine physicians using the PACS tool; these reviewers were blinded to the randomization group. A 60-minute training session on the tool was provided for clarity. In sets of two, videos were assessed by both reviewers and then discussed with the primary investigator to answer clarifying questions and make minor wording edits. Adequate agreement was obtained after six videos, and the remaining videos were split between the two reviewers for review and scoring.

Outcomes

The primary outcome was defined a priori as PACS performance scores at 12 months. Secondary outcomes included change in PACS scores between pre- and post-repeat education assessments, change in PACS scores between post-repeat education assessments and assessments three months later, and overall scores on a knowledge survey taken initially and at 12 months that was developed by the authors to address learning objectives. As not all subjects might have participated in all follow-up sessions, the average scores were based on the number of subjects that participated in each session. Only observations where the assessment form and post-assessment form were complete were included in the analysis.

Randomization and blinding

Participants were block randomized to receive education via RCDP debriefing vs. traditional style debriefing. Sample size was determined based on the number of categorical pediatric interns enrolled in the residency program in June 2021. Based on the repeat education subgroups determined by resident schedules, residents were then randomized to RCDP vs. traditional debriefing via a random number generator. Interns remained in their randomized group when they received a repeat teaching intervention at three, six, nine, or 12 months during the year, depending on their pre-determined residency rotation schedule. Residents were not blinded to their study arm and were told which type of education they would be receiving during the pre-brief to maintain psychological safety with a new teaching method. Video reviewers were blinded to resident education group placement.

Statistical analysis

To determine the inter-rater reliability between individuals using PACS to evaluate videos, 10% of the videos were scored independently by both reviewers. Overall agreement and a Cohen’s kappa were calculated.

Average scores on a resuscitation knowledge quiz were calculated by education group at intern orientation, after initial education, and at 12 months. A two-sample t-test was used to test for a significant difference between the two groups in the change in average quiz score from intern orientation to the end of the study. Equality of variances was determined to be equal prior to the t-test analysis.

Spaghetti plots showing average PACS assessment scores over time by education group were created for each cohort. An additional plot showing average PACS assessment score over time for subjects who received repeat education versus subjects who did not receive repeat education was created, and a t-test was used to compare the mean score between the two groups at 12 months.

A linear mixed effects model was created to examine the relationship between PACS score, time point, and education group (RCDP vs. traditional). The primary outcome was the mean PACS score, and the independent variables included time point, education group, and the interaction of time point and education group. Subject was included as a random effect to account for individual variability and correlation between a subject’s repeated measures.

ANOVA models compared the mean changes in PACS assessment scores between pre- and post-repeat education assessments and between post-repeat education assessments and assessments three months later by education follow-up time and education group.

## Results

Demographics

A total of 34 interns were initially enrolled in the study; one resident opted out of the study. Seventeen residents were randomized to RCDP and 17 to traditional debriefing. Five residents were excluded from the traditional group and one from the RCDP group for lack of subsequent educational and assessment follow-up (Figure 1).

From 28 subjects, there were 124 observations over the study period. Most (96%) interns had previous simulation experience in medical school, including mock codes (93%), but only half reported participation in a simulated code in the previous six months. More than half (54%) reported never experiencing a code on a patient. Most (86%) had performed CPR in simulation; however, only 25% had performed CPR clinically. Nearly half (46%) had performed BMV clinically. Twelve participants were considering a critical care/high acuity fellowship, such as pediatric emergency medicine and pediatric critical care of neonatology.

Inter-rater reliability

Cohen’s kappa coefficients of video reviewer scores showed moderate (0.41-0.6) or substantial (0.61-0.8) agreement on 10 of the 14 items in the PACS tool [26]. Items that showed poor agreement were “identifies hypopnea,” “assessment of pulses and perfusion,” “identifies correct rhythm,” and “resumes compressions immediately after shock or pulse check.” The overall percent agreement for the tool was 70.5%.

Simulation knowledge quiz

Scores on a resuscitation knowledge quiz were similar in both groups at intern orientation. Those in the RCDP group had a larger gain in knowledge after their initial education session, improving from 57% to 71%, and this knowledge was retained with additional improvement to 78% by the end of intern year. Those in the traditional group improved from 55% to 65% after their initial education session. This improvement was retained at 65%, but no further improvement occurred over the year. No statistically significant difference in change in average simulation quiz scores from intern orientation to the end of the intern year was found between the RCDP and traditional groups (p = 0.16).

PACS scores

**Figure 3:**
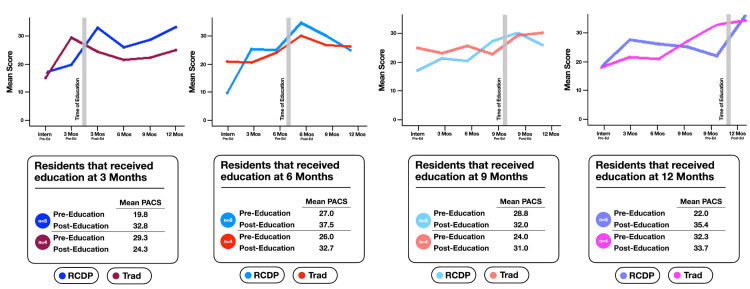
Scores on PACS assessment at quarterly intervals based on re-education subgroup. PACS: pediatric airway and circulation skills; RCDP: rapid cycle deliberate practice; Trad: traditional.

**Figure 4:**
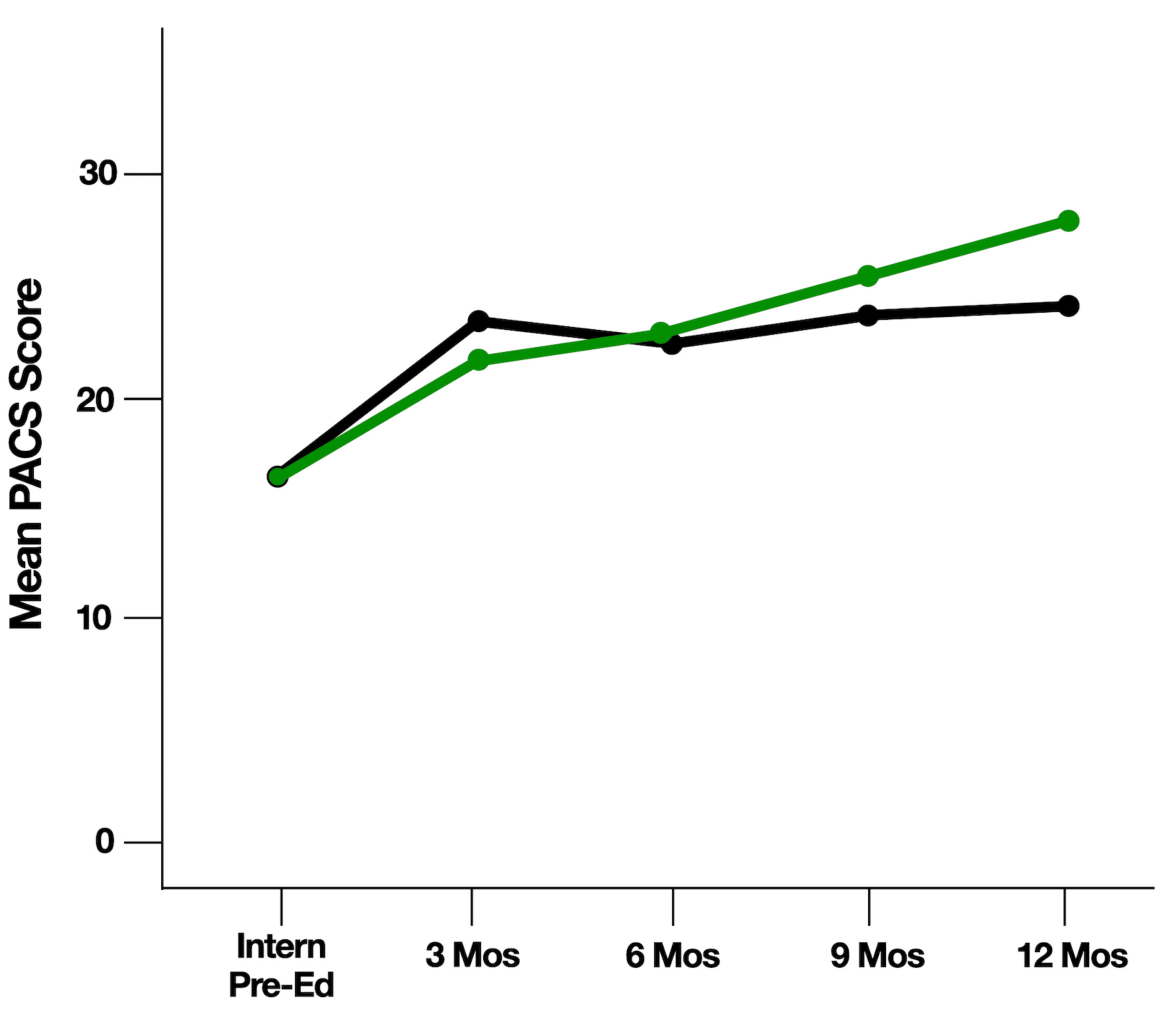
Pediatric airway and circulation skills (PACS) scores in groups that received repeated education compared with the 12-month subgroup that had not yet received repeat education. Green: received repeat education; black: did not receive repeat education.

Graphically, average PACS scores demonstrated improvement over time from the start of the study to the end of the study within each follow-up cohort, irrespective of educational group (Figures 2, 3). Additionally, average PACS scores were higher at 12 months for subjects who received repeat education (n = 19) than for subjects in the 12-month subgroup who had not yet received repeat education (n = 9) (Figure 4); however, this was not statistically significant (p = 0.41).

The linear mixed effects model demonstrated that while PACS scores significantly increased over time for the total cohort, there was no significant difference between educational groups (RCDP vs. traditional debriefing) (Figure 2, middle row).

An ANOVA model demonstrated that within the three-month follow-up time cohort, the RCDP group had a statistically significant improvement in mean PACS score between pre- and post-education assessments when compared with the traditional group. At the three-month post-education session, the RCDP group had a mean PACS score of 32.8 compared with a pre-education mean PACS score of 19.8. Meanwhile, the mean PACS score among members of the traditional group at the post-education session was 24.3, down from a mean pre-education score of 29.3. However, there were no statistically significant differences between groups in the other time cohorts of repeat education at six, nine, or 12 months (Figure 3).

A second ANOVA model compared scores post-repeat education to scores three months later to assess retention and showed no significant difference between educational groups or by timing of repeat education.

## Discussion

This study attempted to address whether RCDP debriefing was better than traditional style debriefing for overall skills acquisition and skills retention in a repeated-measures study design. We found no statistically significant difference in overall skills acquisition or retention of skills from intern orientation to 12 months when comparing RCDP to traditional debriefing. This may have been secondary to a lack of power, given the fixed number of residents. It may have been secondary to the significant confounders of simulation education that occurred throughout the year, such as clinical experience, in situ simulation education, acuity of scheduled rotations, and seasonality of high acuity rotations. This may have diluted the effect of RCDP training, which did show a significant improvement in the three-month cohort. There was significant improvement in overall scores across education groups, suggesting that simulation-based resuscitation education, including RCDP, is effective as previously demonstrated [27]. Additionally, mean PACS scores in groups that had received repeat education were higher, although not significant, than those who had not yet received repeat education, suggesting that repetition of resuscitation education improves skill.

Timing and immediate skill acquisition: RCDP vs. traditional debriefing

We demonstrated improvement in mean PACS scores from pre-repeat education to post-repeat education in the RCDP group at each time point, with the three-month follow-up cohort showing significantly greater score increases in the RCDP group than in the traditional group. This suggests that RCDP may be superior to traditional debriefing for skills acquisition immediately after an educational session at one specific time point. RCDP superiority did not persist at other time points of repeated education or in overall improvement based on the linear mixed effects model, which demonstrated that PACS scores generally improved over time; however, these score improvements were not dependent on education type. Overall, our results are similar to prior studies, although our aim was to assess overall resuscitation skills acquisition as opposed to individual, isolated skills. A scoping review of RCDP assessed 23 articles on RCDP and showed improved skills performance post RCDP education sessions [23]; however, this review was not able to demonstrate clear superiority of RCDP when compared with more traditional forms of debriefing. Our study demonstrated RCDP's superiority only within the cohort that received repeat education at three months. This is consistent with a study by Sullivan et al., demonstrating three months as the most appropriate timing for repeated basic life support education [28]. We postulate that this may be due to the rapidity of repeat education received by this specific group and the lack of other confounding simulation sessions that occur during the year in other units and within other areas of the simulation curriculum; however, this is speculative. This suggests that RCDP debriefing may be more beneficial sooner after initial education, while traditional debriefing appears to be of similar value as time from initial education increases.

Skills retention: RCDP vs. traditional debriefing

Similar to prior studies, there remains uncertainty whether RCDP is superior for retention of skills [29,30]. Our study demonstrated no significant difference in PACS scores three months post educational intervention when comparing RCDP and traditional debriefing groups, suggesting that RCDP is not superior to traditional debriefing for retention of skills at three months. Surapa Raju et al. compared RCDP to plus/delta debriefing styles with a nine-month “booster” education session and did not demonstrate a significant difference in retention of skills between the groups at three months, suggesting similar results to ours [20]. This study also had a small sample size (n = 28), similar to other simulation education studies that are limited by a fixed number of learners. Won et al. demonstrated improved odds of defibrillation in pediatric residents who received RCDP simulation training one to 12 months prior, when compared to residents who received post-simulation debriefing, defined similarly to our definition of traditional debriefing [31]. While the study showed improved retention of the specific skill of time to defibrillation in RCDP-trained residents, it assessed one specific skill as opposed to a composite of multiple resuscitation skills.

Pediatric resuscitation skills are integral to residency training, and some are included in the ACGME pediatric training requirements. More specifically, for those residents entering critical care fields, such as emergency medicine, intensive care, or rural general pediatrics, having a strong skill set in basic airway resuscitation and CPR is necessary for improved care. However, the ACGME recently limited the number of procedural competencies that are required for graduation, many of which are specific to resuscitation care. In a recent editorial by Nawathe et al., a team of esteemed simulation educators made a call to action to the ACGME Task Force to better leverage simulation-based educational practices to achieve pediatric procedural skills competency, given that clinical opportunities are not sufficient [32]. It has been demonstrated that the ways in which we are teaching residents resuscitative care are not effective when we use PALS certification training programs alone [33]. However, it is still unclear what type of additional education is necessary that is both effective but also considers facility, facilitator, and program resources. Simulation-based training seems to be the most appropriate, given the low frequency of these events; however, the optimal length, style, and frequency of educational sessions are still being elucidated. Ericsson’s theory of learning mastery is centered on the concept of deliberate and repetitive practice with expert feedback [13]. RCDP simulation debriefing is founded on this learning theory and aims to provide a superior method of teaching resuscitation skills. While this study did not have the power to demonstrate a significant difference between RCDP and traditional debriefing, we were able to demonstrate a robust repeated-measures study design in an attempt to assess differences in the timing of education. This study design has the potential to demonstrate significance and shed light on this question if powered appropriately, in particular, the suggestion that RCDP may be superior for repeat teaching, specifically at three months.

Limitations

There are several limitations to our study. First, although the PACS tool was based on validated tools to review simulations, we found moderate agreement between reviewers on most items, while some critical items showed poor agreement. Items with poor agreement may have been more challenging to assess via video, such as “assesses perfusion” or “identifies hypopnea,” as the reviewer would rely on the resident to verbally state what they were assessing, as opposed to seeing a physical action, which implied they had performed this assessment. This may represent an inherent flaw in the application of the tool and may need to be addressed in the future for greater agreement. Additionally, poor inter-rater reliability may be secondary to the inherent challenges of interpreting actions and/or intentions via video review, as some specific steps may be missed by a reviewer if the participant did not speak them out loud. Second, our study size was limited to the number of intern residents in the entering class, creating small subgroups within each quarterly educational session. These numbers were further reduced when residents were lost to follow-up in subsequent sessions. There is an inherent participation bias in voluntary surveys for the knowledge assessment, which may have affected those results. Third, while all interns rotate through the same units and have the same overall simulation curriculum, their educational experiences depend on many uncontrollable factors, such as seasonality of high acuity rotations, ability to participate in in situ simulation sessions, and varying autonomy provided by attending physicians, which may have led to changes in their performance. Additionally, the timing of repeat education was not randomized but based on the resident schedule, which may have introduced further bias. Fourth, our aim was to assess overall resuscitation skills using a comprehensive assessment. However, had we considered individual skill performance, there may have been significant differences in isolated skills, demonstrating specific areas in which RCDP may be most helpful. Lastly, due to the restraints of intern orientation scheduling, we were not able to assess residents immediately after their initial education at intern orientation, and therefore do not have data on the immediate effects of the intervention prior to any residency training and experience.

However, given these limitations, our study was conducted in a real-world scenario and had many strengths. Selection bias was minimal due to the inclusion of all residents and the randomized nature of delivering RCDP versus traditional education. Although there was a loss to follow-up in subsequent simulation sessions, the proportion of residents between RCDP and traditional cohorts who were lost to follow-up was similar (6/17 vs. 4/17, respectively). Lastly, we were able to investigate the benefit of RCDP versus traditional education at multiple time points to determine short-term and long-term overall resuscitation skill acquisition.

## Conclusions

This longitudinal, randomized, stepped-wedge study comparing RCDP to traditional debriefing demonstrated improved overall resuscitation skills at 12 months in the overall cohort, but no difference between debriefing techniques or among timing of repeat education. RCDP continues to show trends toward improved skills acquisition, significant only at the three-month repeat education time point, but no persistent benefit at three months after the educational intervention. Given the challenges to studying Kirkpatrick level 3 and 4 outcomes in pediatric trainee resuscitation skills, it may be useful to gain more qualitative information from pediatric trainees regarding their experiences with different styles of simulation-based resuscitation training and clinical resuscitation experience. Alternatively, a multi-site randomized trial similar to this one would yield a higher power, allowing for an opportunity for significance and also allowing for more generalizability of results. Additionally, a gold standard of RCDP implementation (timing of pauses, standardized pre-brief, number of objectives, as examples) would allow for improved assessment of the superiority of teaching styles. While this study demonstrates improved scores in the RCDP group at one time point, the true superior training type is still uncertain and requires further investigation, preferably with a large sample size from multiple institutions.

## Appendices

**Table 1:** Simulation scenarios. BMV: bag-mask ventilation; CPR: cardiopulmonary resuscitation; HR: heart rate; RR: respiratory rate; BP: blood pressure; SpO2: oxygen saturation; PICU: pediatric intensive care unit; THC: tetrahydrocannabinol; NC: nasal cannula; PIV: peripheral intravenous; RA: room air; AMS: altered mental status; EMS: emergency medical services; RT: respiratory therapist; NIPPV: noninvasive positive pressure ventilation; CR: capillary refill; AED: automated external defibrillator; VT/Vfib: ventricular tachycardia/ventricular fibrillation; IV/IO: intravenous/intraosseous; ROSC: return of spontaneous circulation.

Section	Details
Scenario	SimBaby: Hypoventilation due to THC overdose. Authors: Shannon Flood, MD & Kathryn Walsh, MD
Objectives	1. Respond to clinical scenario appropriately: a. Basic patient assessment (response, pulses, airway). b. Identify hypoxemia. c. Apply oxygen (NC/simple mask). d. Identify inadequate RR/ventilation. e. Initiate BMV appropriately. f. Problem-solve bagging (airway opening, 2-person technique, adjuncts). g. Monitor for improvement (HR, RR, chest rise). 2. Call for help
Room setup	- SimBaby in crib, not on monitors - PIV in place - Oxygen available in room (NC, simple mask) - Clear box with self-inflating bag - Code cart outside room (contains flow-inflating bag) - 10 kg EPIC code sheet available
Initial vitals	RR: 14, Temp: 37°C, HR: 110, BP: 86/54, SpO₂: 88% (RA). Patient wakes to painful stimuli with whimpering
Scenario background	6-month-old female, 10 kg, presented for AMS. EMS report: sleepy, improved slightly, given blow-by O₂. Gummies found at the scene.
Stops & progression	Hard stop: Identification of roles. Hard stop: Identify low RR, cyanosis, hypoxemia, ↓ alertness. Soft stop: Apply O₂ via NC. Stage 1: RR 6, HR drifts to 64, SpO₂ = 78% RA. Soft stop: Call for help. Hard stop: BMV teaching. Soft stop: Airway suctioning/positioning. Stage 2: After BMV → HR 120s, SpO₂ = 96%. Soft Stop: Narcan Stage 3: After Narcan → No change. Hard stop: Shared mental model: THC ingestion, supportive care, call RT for NIPPV
Teaching points	Soft stops = Pause & resume at the same point. Hard stops = Rewind to start or last hard stop. Rotate roles at each stop for hands-on airway practice
Expected interventions	1. Identify roles. 2. Assess responsiveness. 3. Check pulses. 4. Place on monitor & assess respirations. 5. Identify hypoxemia & cyanosis, share mental model. 6. Place oxygen. 7. Recognize inadequate respirations & share mental model. 8. Begin BMV. 9. Call for help. 10. Provide adequate BMV (1-sec insufflations, chest rise, 3–5 sec interval). 11. Identify airway adjuncts (2-person bag, oral/NP airway, suction). 12. Understand self-inflating vs. flow-inflating bag. 13. Share mental model again. 14. Consider Narcan

Section	Details
Scenario	VT/VFib arrest: SimMan Sepsis. Authors: Shannon Flood, MD & Kathryn Walsh, MD
Objectives	1. Respond to the clinical scenario appropriately. a. Assess patient (response, pulses, airway). b. Identify no pulse. c. Start compressions immediately (“No pulse, starting compressions”). d. 2nd provider joins in airway role, coordinates 30:2 compressions:breaths. e. The airway provider ensures chest rise. f. 3rd/4th providers place backboard & Zoll pads. g. Identify V-tach w/o pulse. h. Deliver 120 J shock, resume compressions. i. 2-min timer set. j. Shared mental model repeated. k. Rhythm check: Vfib → 150 J shock. l. Repeat shared mental model, review Hs/Ts m. Next rhythm check: Sinus rhythm w/pulse
Room setup	- High-fidelity SimMan on CR monitor, pulse ox, BP cuff attached (no BP reading) - Normal pupils - PIV in place (no IO) - Code cart outside room - Code sheets available
Initial vitals	HR: 245, VT, RR: 0, BP: 0, SpO₂ not picking up, patient unresponsive
Scenario background	16-year-old male admitted for pneumonia/flu. Mother alerts staff that the patient is not responding. Weight 65 kg.
Stops & progression	Hard stop: Identify unresponsive patient & call for help. Hard stop: Identify no pulse & start compressions. Soft stop: Backboard, stool. Hard stop: Place Zoll pads (review peds vs. adult pads, AED vs. defibrillator mode). Hard stop: Identify V-tach, shock at 120 J, share mental model. Soft stop: Obtain IV/IO access, labs. Soft stop: Set 2-minute timer, switch compressors, emphasize CPR quality. Stage 1: After 1st shock → Vfib persists, no pulse. Soft stop: Administer epinephrine. Hard stop: Identify VFib, shock at 150 J, share mental model & Hs/Ts. Stage 2: After 2nd shock → ROSC, HR = 130, BP = 84/50, RR = 6, SpO₂ = 92%, wakes to pain. Hard stop: Identify ROSC, assess spontaneous respirations, BP, labs, ECG, notify PICU/renal, consider intubation
Teaching points	Soft stops = pause & resume. Hard stops = rewind to start or last hard stop. Rotate roles for practice in compressor/Zoll roles
Expected interventions	1. Identify roles. 2. Assess responsiveness & call for help. 3. Check pulses & start compressions. 4. Begin two-person CPR (30:2). 5. Place backboard & Zoll pads. 6. Identify V-tach as a shockable rhythm. 7. Deliver 120 J shock, resume CPR. 8. Continue CPR x 2 min with good compressions. 9. Obtain IV access, draw labs, and prepare epinephrine. 10. Identify VFib, deliver 150 J shock, resume CPR. 11. Review Hs/Ts & treat hyperkalemia if indicated. 12. Identify ROSC, reassess patient, plan ICU transfer.

**Table 2:** Resuscitation knowledge quiz. CPR: cardiopulmonary resuscitation; CPAP: continuous positive airway pressure; FiO_2_: fraction of inspired oxygen.

#	Question	Options
1	What is true about BAG-MASK ventilation?	- A self-inflating bag can provide CPAP when connected to an oxygen source - A self-inflating bag can only be used when not connected to an oxygen source - A flow-inflating bag can be used when not connected to an oxygen source - A self-inflating bag can provide rescue breaths at 21% FiO₂ when not connected to an oxygen source
2	In an infant patient receiving CPR with an advanced airway, what is the rate of breaths the patient should receive?	- 4–6 breaths per minute (1 breath every 10–12 seconds) - 8–10 breaths per minute (1 breath every 6–8 seconds) - 12–14 breaths per minute (1 breath every 4–5 seconds) - 20–30 breaths per minute (1 breath every 2–3 seconds)
3	How do you measure the size for an oropharyngeal airway?	- Middle of mouth to tragus of the ear - Tip of nose to tip of the earlobe - Corner of mouth to angle of the jaw - Tip of the nose to angle of the jaw
4	How do you measure the size for a nasopharyngeal airway?	- Middle of mouth to tragus - Tip of nose to tip of the earlobe - Center of mouth to angle of the jaw - Tip of the nose to angle of the jaw
5	How deep should your compression be in quality CPR on a child?	- 1/3 the anterior-posterior diameter of the chest (1.5–2 inches) - 1/4 the anterior-posterior diameter of the chest (1–1.4 inches) - 3 centimeters - 2–2.4 inches
6	What is the ratio of compressions to breaths in two-person CPR without an advanced airway in a pediatric patient?	- 15:2 - 30:2 - 15:1 - 30:4
7	At what dose should the first shock be delivered to a patient in cardiac arrest with a shockable rhythm?	- 1 Joule/kilogram - 2 Joules/kilogram - 4 Joules/kilogram - 5 Joules/kilogram
8	When should you place the Zoll defibrillator in Analyze mode?	- The Zoll defibrillator should never be placed in Analyze mode if a healthcare professional is leading the resuscitation - Prior to giving any shock to the patient in order to determine if it is a shockable rhythm or not - Only if you cannot feel a pulse - If the patient has pulseless electrical activity, the defibrillator should be placed in Analyze mode
9	What is an example of a shockable rhythm?	- Ventricular fibrillation without a pulse - Ventricular tachycardia with a pulse and good perfusion - Asystole - Pulseless electrical activity (PEA)
10	What would be an appropriate rhythm to give a synchronized cardioversion shock?	- Unstable supraventricular tachycardia - Ventricular tachycardia with a pulse and poor perfusion - Both a and b - None of the above
11	What is the dose of IV epinephrine in cardiac arrest resuscitation?	- 10 mg/kg - 1 mg/kg - 0.1 mg/kg - 0.01 mg/kg
12	How often can IV epinephrine be repeated in a cardiac arrest resuscitation?	- Every 1–2 minutes - Every 2–3 minutes - Every 3–5 minutes - Every 6–8 minutes
13	Which is true about performing high-quality CPR?	- Minimize interruptions in chest compressions to <30 seconds - Give continuous compressions at a rate of 80/min - Check pulse immediately after delivering a defibrillation shock - Each rescue breath should result in visible chest rise

**Table 3:** Pediatric Airways and Circulation Skills (PACS) checklist. BMV: bag-mask ventilation; PALS: pediatric advanced life support; HR: heart rate; RR: respiratory rate; BP: blood pressure; BS: breath sounds; NC/SFM: nasal cannula/simple face mask; VS: vital signs; VT: ventricular tachycardia.

Step	Description	Score criteria
1. Initial assessment of responsiveness & airway patency	Establish if the patient is phonating, check for an open airway, and identify the protection of the airway	3: States patient is crying/airway intact within 30 sec. 2: Performs >30 sec. 1: Performs but does not state crying/look for obstruction. 0: Does not perform
2. Assessment of breathing adequacy	Auscultation, work of breathing, adequacy of ventilation. Facilitator: Rales diffusely, decreased BS in bases, pan retractions	3: Assesses breathing <1 min, listens to all lung fields. 2: Assesses >1 min. 1: Does not listen to all lung fields/ask about retractions. 0: Does not perform
3. Initiate appropriate initial breathing support	Places supplemental oxygen, ensures effectiveness	3: Oxygen <30 sec after resp assessment, NC/SFM correctly placed. 2: Oxygen >30 sec, but correctly placed. 1: Incorrect placement. 0: Does not perform
4. Identifies hypopnea/hypoventilation, starts BMV	States the need for additional respiratory support, and obtains an appropriately sized mask	3: States need + obtains mask <30 sec after VS reassessment. 2: States need + obtains >30 sec. 1: Wrong size mask/incorrect. 0: Does not state the need or obtain a mask
5. Supports ventilation with adequate BMV	Correct placement, seal, technique, chest rise	3: Adequate BMV <45 sec after VS reassessment. 2: Adequate BMV >45 sec. 1: Wrong size, poor seal, poor technique, no chest rise, wrong rate. 0: Does not perform
6. Calls for help	Activates help appropriately	3: Calls <30 sec after BMV start. 2: Calls <1 min. 1: Calls >1 min. 0: Never calls
7. Reassess patient after BMV	HR: 192, RR: (bagging rate), BP --/--, O2 sat -, Rhythm: VT	3: Reassesses VS <30 sec after BMV. 2: Reassesses VS >30 sec. 0: Does not perform
8. Assessment of pulses & perfusion	Pulse check within 10 seconds of recognizing no BP	3: Pulse check <10 sec. 2: Pulse check >10 sec. 1: Does not recognize no BP. 0: Does not perform
9. Initiates compressions	Starts chest compressions appropriately	3: Starts <10 sec after no pulse. 2: Starts >10 sec. 1: Does not feel pulse. 0: Does not perform
10. Adequate chest compressions	Correct ratio, rate, minimal interruptions	3: <10 sec off chest for pulse checks, 30:2 ratio, 100–120/min. 2: One area not adequate. 1: >1 area not adequate. 0: Does not perform
11. Places pads on the patient	Pads correctly placed	3: <1 min after compressions started. 2: >1 min after compressions started. 1: Incorrect pad placement. 0: Does not place pads
12. Identifies rhythm, states the correct PALS algorithm	Identifies rhythm, verbalizes correct algorithm	3: Within 30 sec. 2: One area inadequate. 1: >1 area inadequate
13. Defibrillate patient (if necessary)	Orders correct joules, clears, and shocks appropriately	3: <30 sec after pad placement. 2: >30 sec after pad placement. 1: Pads incorrect. 0: Fails to shock when indicated or shocks a non-shockable rhythm
14. Resume compressions immediately	After a shock or pulse check	3: <5 sec after shock/pulse check. 2: >5 sec after shock/pulse check. 1: Does not start compressions. 0: Does not perform
